# Obesity-Related Factors in Turkish School Children

**DOI:** 10.1100/2012/353485

**Published:** 2012-04-01

**Authors:** Cihad Dündar, Hatice Öz

**Affiliations:** ^1^Department of Public Health, Faculty of Medicine, Ondokuz Mayıs University, 55139 Samsun, Turkey; ^2^Provincial Directorate of Health, 55060 Samsun, Turkey

## Abstract

*Objective*. To determine the prevalence of obesity and its risk factors in Turkish children. *Method*. This cross-sectional survey was conducted on students including 1271 boys and 1206 girls selected from 20 secondary schools in Samsun, Turkey. A predesigned questionnaire was used to elicit the information on individual characteristics. The height and weight of students were measured in their classroom. Obesity was defined as body mass index at or above the 95th percentile for age of the sex-specific CDC growth charts. *Result*. The mean age was 12.8 ± 0.9 years, and the prevalence of obesity was found at 10.3%. There were higher numbers of obese students in boys than in girls (*X* ^2^ = 53.4; *P* < 0.001). The prevalence of obesity was 10.0% and 16.8% in public and private school students, respectively. The percentage of obese children in students who skipped breakfast was found to be higher than that in the group that consumed 3 meals a day regularly. There was no difference at time spent in sedentary behavior except watching TV, and prevalence of obesity in the group of students watching television over 3 hours per day was higher than that in their counterparts (*X* ^2^ = 13.6; *P* < 0.01). The time of engagement in sports was lower in obese group statistically (*F* = 8.9; *P* < 0.001). *Conclusion*. In order to prevent childhood obesity, monitoring children's lifestyle by parents is necessary.

## 1. Introduction

The issue of being overweight and obese is becoming an increasingly prevalent problem in both the developed and developing world, and it is one of the most serious public health challenges of the 21st century [1]. In 2007, it was estimated that globally 22 million children under 5 years were overweight, with more than 75% of overweight and obese children living in low- and middle-income countries [2]. It is not only the scale of childhood obesity that is challenging, but also the speed at which the prevalence has increased. The greatest annual increases in obesity since 1970 in school children have been in North America and Western Europe [3].

Obesity, which is defined as the condition of excessive fat in the body, has significant health consequences. It is the result of undesirable weight gain caused when people consume more energy than they expend [4]. The explanation of the primary cause of being overweight and obese is not clear in adolescents, although dietary, genetic, and physical activity patterns must be important factors [5]. It is seen as a problem associated with adulthood; obesity during childhood is also becoming a concern. However, childhood obesity is an important public health issue with increasing prevalence and important consequences (comorbidities of obesity in childhood include type 2 diabetes, hypertension, dyslipidaemia, emotional and behavioural problems, asthma, and sleep apnea) [6].

Many survey data indicate that leisure activity is increasingly sedentary, due to the wide availability of entertainment such as television, videos, and computer games. This sedentary behavior is emerging as an important component of obesity and should be recognized as a behavior that is distinct from physical activity [7]. In addition, with increasing urbanization, there has been a decrease in the frequency and duration of physical activities of children, such as walking to school [8, 9].

The aim of this study is to determine the prevalence of obesity and the related factors in a representative sample of secondary school children living in the province of Samsun, the 15th high populated city of Turkey.

## 2. Methods

### 2.1. Samples and Survey Procedures

This cross-sectional regional survey was conducted on 2477 students selected from classes VI, VII and VIII in a total of 20 secondary schools in the province of Samsun in April 2009. This representative sample was selected from 24.691 students in 68 secondary schools by a multistage random sampling method according to school type (public or private), number of classes, and gender. In choosing the sample, the following weighing procedures were used. First, the number of private and public schools was determined according to their percentages in all secondary schools in central Samsun. Hereafter, the number of classes VI, VII and VIII in the different schools was determined according to their percentages in all secondary schools in central Samsun. Finally, the classrooms were chosen randomly. The participants filled out the predesigned questionnaires under the supervision of trained nurses. The height and weight of students were measured in the classrooms. For the measurements, the students were dressed in light indoor clothing and had bare feet or stockings. The subjects were weighed to the nearest 0.1 kg with an electronic scale (Bosch, PPW4010; Stuttgart, Germany) that was calibrated daily at the beginning of each working day. Height was measured to the nearest 0.1 cm with a stadiometer (Charder HM200P Portstad Portable Stadiometer, Stuttgart, Germany) in a vertical erect position, with parallel feet, and with the shoulders and bottom touching the wall. The nurses recorded birth date and measured height and weight twice; the averages were recorded. The height and weight data were used to calculate the BMI (kg/m^2^) using the formula: weight (kg) divided by height (m) squared. The cut-off points of CDC growth charts and percentiles of the age- and gender-specific BMI were used to determine Turkish children's underweight, overweight, and obesity status [10]. Students who had BMI for age ≥85th and <95th percentile of the reference population were classified as overweight and BMI for age ≥95th percentile of reference population were classified as obese. Students who had BMI for age <5th percentile of the reference population were classified as underweight.

### 2.2. Sedentary Behaviour

Television, videos, and computers are well-recognized domains of sedentary behaviour, but other sedentary activities in which adolescents engage include studying, sitting around and talking with friends, travel by motor vehicles, hobbies, and crafts. The questionnaire was developed to measure a broad range of sedentary activities common amongst adolescents reported in the literature [9]. The students were asked to report the time usually spent watching television, videos, or playing video games; using a computer for fun or study; doing homework/study or reading for fun; talking on the telephone, sitting with friends or hanging out; travelling in a car, bus, or train, before and after school on a usual weekday and for each weekend.

### 2.3. Statistical Analysis

Data was analyzed using SPSS for Windows, Release 12.0 (SPSS, Chicago, Ill, USA). Logistic regression and Chi-square analysis were applied to determine the statistical significance of the difference in the prevalence of obesity between the groups with respect to gender, school type, and time spent in sedentary behaviour. The significance of differences was defined as *P* < 0.05.

The study was approved by Local Ethic Committee of Medical Research of Ondokuz Mayıs University, and written informed consent was obtained from each parent.

## 3. Results

The sample is composed of 2477 children, including 1271 boys (51.3%) and 1206 girls (48.7%), 11 to 14 years old, attending secondary schools in the province of Samsun. The mean age was 12.8 ± 0.9 years, and the prevalence of obesity was defined at 10.3% (Table 1).

As shown in Table 2, there were higher numbers of overweight and obese students in boys than in girls statistically (*X* ^2^ = 53.4; *P* < 0.001).

A total of 95.9% of students attended public schools, while 4.1% attended private schools. The prevalence of obesity was 10.0% and 16.8% in public and private school students, respectively. The difference was statistically significant (*X* ^2^ = 4.9; *P* < 0.05).

As presented in Table 3, 46.8% of students had eaten their 3 daily meals regularly every day. The proportion of obese students that consumed fewer than three meals was significantly higher than that of those who consumed meals regularly during the day (*X* ^2^ = 16.2; *P* < 0.01).

Further, the number of students who skipped breakfast was 731 (29.5%), and the prevalence of obesity in students who skipped breakfast was found to be higher than that in their counterparts (*X* ^2^ = 9.1; *P* < 0.05).

While underweight students reported that they spend 5.2 ± 4.2 hours doing sports a week, normal, overweight, and obese students reported that they spend 6.0 ± 4.7 hours, 5.8 ± 4.6 hours, and 4.3 ± 3.5 hours, respectively. The difference was highly significant (*F* = 8.9; *P* < 0.001). 

As shown in Table 4, the time spent in sedentary behaviour was the same in all BMI groups.

The prevalence of obesity was found to be higher in the group that spent over 3 hours watching TV than in those that spent less time (Table 5). 

Step-down multiple logistic regression using backward LR method was applied to determine the significant correlates of obesity in the study population. The final model showed that gender (boys: OR = 1.557; 95% CI 1.174–2.064), irregular meal habits (OR = 1.843; 95% CI 1.398–2.430), private school (OR = 2.230; 95% CI 1.384–3.593), and sport (OR = 0.900; 95% CI 0.867–0.934) were significantly associated with obesity among the study population. The area under the curve for this final model was derived by multivariate logistic regression and was found to be 89.7% suggesting a good fit of the model.

## 4. Discussion

Childhood obesity is rapidly increasing in Europe, with the highest prevalence rates (31–39%) of overweight children reported in Mediterranean countries and in the USA (30% of children and adolescents who age 5–17 years are overweight) [11]. In this study the overall prevalence of obesity in secondary school children aged 11 to 14 years was found to be 10.3% and the prevalence of overweight was 22.4%. The prevalence of obesity is lower than in the USA and Europe but has demonstrated an increase in BMI amongst adolescents in the last decade. By comparison, in 1998, the prevalence of obesity in school children aged 12 to 15 years was found to be 5.1% in the province of Samsun [12]. High consumption of foods rich in fat and calories and the sedentary lifestyle among communities in the East Mediterranean region, in combination with a shift from traditional food to more Westernized foods characterized by high fat, high cholesterol, high sodium, and fibre contents, seem to be playing an important role in the rise of the level of obesity.

In some recent researches it was found that, of the obese and overweight children, most belonged to high social class families, and that less belonged to middle and low social class families [13, 14]. In our study, the prevalence of obesity was 10.0% and 16.8% in public and private school students, respectively. Similar findings were reported from India, that is, 6% were in corporation schools and 24% in well-off schools [15].

Consistent with some reports [16–18], and contrary to others [19, 20], the prevalence of obesity was found significantly higher in boys than in girls. The population living in the province of Samsun, in Northern Turkey, has middle-upper socioeconomic status. While the higher prevalence of obesity in boys occurring in Western Turkey has a high socioeconomic status, the prevalence of obesity is higher in girls in Eastern Turkey who are from a low socioeconomic status. For example, the prevalence of obesity was found to be 0.9% in boys and 3.8% in girls, aged between 8 and 17 years, in Elazig, in Eastern Turkey; contrary to these low prevalence rates, Neyzi et al. found a higher prevalence of obesity as 11.2% in adolescent boys and 9.4% in adolescent school girls aged 9–17 years in Istanbul, in Western Turkey [21]. Similarly, the prevalence of being overweight and obese was found to be higher in boys than in girls in Brazilian [22], Canadian [23], and Australian [24] adolescents. Unlike these results, in the studies conducted on Tehrani [18] and Egyptian [25] adolescent students, the prevalence of being overweight among girl students was found higher than among boys. A potential explanation could be that girls who live in developed countries have more chance with outdoor activities and sports than those who live in undeveloped and in some developing countries. Since the difference in obesity cannot be explained by differences in socioeconomic status alone, additional research is required to clarify why these regional and gender differences exist.

The prevalence of obesity was found to be significantly higher in the group that ate their daily meals irregularly when compared to the group that consistently ate their 3 daily meals. Previous studies have shown an inverse association between meal frequency and the prevalence of obesity in children [26] while others failed to detect significant associations [27]. Some studies have suggested that skipping breakfast is associated with the overweight/obese status [28, 29]. Skipping breakfast was also found to be associated with increased BMI in the current study. Moreover, it should be taken into consideration that the regression analysis indicated that irregular meal consumption is seen as a predictor of being obese. This highlights the importance of promoting the consumption of breakfast and all other meals throughout adolescence.

Although some studies found a better pattern of eating habits in children engaged in sports compared with their nonactive peers [30], in a current study both meal frequency and skipping breakfast were not associated with physical activity. Furthermore, this data needs some further attention since a significant association was found between meal frequency and obesity which linked the breakfast skipping behaviour among children in developed countries. Ekelund et al. [31] reported that they did not observe any association between TV viewing and physical activity, and the association between TV viewing and adiposity was independent of physical activity. This may suggest that TV viewing does not displace physical activity and that other factors, such as dietary behaviour and quality while viewing TV, may influence energy balance and thereby body weight. In Turkish adolescents, the prevalence of obesity was found to be higher in the group that spent more than 3 hours watching TV than in those that spent less time, and there was no difference in the time spent in sedentary behaviour among different BMI categories except the duration of watching TV. Some researchers reported that children who spent more time watching television per day had significantly greater BMI, compared with those watching less than 2 hours per day [7, 32]. One meta-analysis, which found that sedentary behaviour was not associated with physical activity or BMI, also suggests that future research needs to consider different types and levels of sedentary behaviours when examining associations between physical activity and obesity [33]. The relationship between BMI and physical activity may be moderated by sedentary activity. This may explain why some researchers did not find an association between BMI and physical activity in children and adolescents despite the postulated relationship between reduced energy expenditure and obesity.

In conclusion, the current data provides strong evidence that supports the idea that the amount of hours spent watching television and irregular meal consumption of adolescents are directly related to the risk of obesity. In order to prevent childhood obesity, it is the duty of parents to monitor their children's lifestyle choices by reducing the consumption of fast food, being involved in regular family meals, and limiting the number of hours spent by their children watching television. In addition, in order to avoid greater pressure on future healthcare-related issues caused by obesity-related disorders, it is imperative to develop effective prevention strategies, for either all children or simply those Turkish adolescents living in urban areas who tend to be at a higher risk of obesity.

## Figures and Tables

**Table 1:** Demographic characteristics of study group.

	Boys (*n* = 1271)		Girls (*n* = 1206)		Total (*n* = 2477)	
Characteristics	Mean ± Sd	Range	Mean ± Sd	Range	Mean ± Sd	Range
Age, y	12.9 ± 1.0	11–14	12.8 ± 0.9	11–14	12.8 ± 0.9	11–14
Height, cm	155.2 ± 10.5	120.0–193.0	154.5 ± 7.5	128.0–177.0	154.9 ± 9.2	120.0–193.0
Weight, kg	48.6 ± 12.8	21.2–103.9	48.5 ± 11.3	22.8–97.6	48.5 ± 12.1	21.2–103.9
BMI, kg/m^2^	19.9 ± 3.7	12.4–40.0	20.2 ± 3.9	11.9–40.5	20.1 ± 3.8	11.9–40.5

**Table 2:** BMI categories by gender.

	Gender			
BMI categories	Boys		Girls	
	*n*	%	*n*	%
Underweight	26	2.0	45	3.7
Normal	753	59.2	845	70.1
Overweight	354	27.9	200	16.6
Obese	138	10.9	116	9.6

Total	1271	100.0	1206	100.0

*X* ^2^ = 53.4; *P* < 0.001.

**Table 3:** BMI categories by daily meal regularity.

BMI categories	Regular (3 times/day)		Irregular	
	*n*	%	*n*	%
Underweight	33	2.9	38	2.9
Normal	765	65.9	833	63.2
Overweight	273	23.5	281	21.4
Obese	89	7.7	165	12.5

Total	1160	100.0	1317	100.0

*X* ^2^ = 16.2; *P* < 0.01.

**Table 4:** BMI categories by time spent in other sedentary behaviour.

Time spent (hours/week)					
BMI categories	PC games and internet*	Homework reading*	Hanging out*	Travel*	Cinema*
Underweight	5.7 ± 4.3	8.2 ± 6.4	4.1 ± 3.8	0.9 ± 0.6	0.9 ± 0.8
Normal	5.8 ± 3.1	7.8 ± 6.5	4.3 ± 3.1	1.3 ± 0.8	1.2 ± 0.6
Overweight	6.3 ± 4.2	8.1 ± 6.7	3.9 ± 2.5	1.4 ± 1.1	1.3 ± 0.3
Obese	6.1 ± 4.6	8.3 ± 7.4	3.7 ± 2.8	1.3 ± 0.9	1.2 ± 0.6

*P > 0.05.

**Table 5:** BMI categories by time spent in watching TV.

	Duration (hours/day)			
BMI categories	<3 hours		≥3 hours	
	*n*	%	*n*	%
Underweight	40	2.7	31	3.2
Normal	1007	67.0	591	60.6
Overweight	323	21.5	231	23.7
Obese	132	8.8	122	12.5

Total	1502	100.0	975	100.0

*X* ^2^ = 13.6; *P* < 0.01.
